# Non-Life Threatening Maternal Morbidity: Cross Sectional Surveys from Malawi and Pakistan

**DOI:** 10.1371/journal.pone.0138026

**Published:** 2015-09-21

**Authors:** Shamsa Zafar, Rachel Jean-Baptiste, Atif Rahman, James P. Neilson, Nynke R. van den Broek

**Affiliations:** 1 Health Services Academy, Islamabad, Pakistan; 2 Oxford Epidemiology Services, Oxford, United Kingdom; 3 Department of Mental Health and Well-Being, University of Liverpool, Liverpool, United Kingdom; 4 Department of Women’s and Children’s Health, Institute of Translational Medicine, University of Liverpool, Liverpool, United Kingdom; 5 Centre for Maternal and Newborn Health, Liverpool School of Tropical Medicine, Liverpool, United Kingdom; The Hospital for Sick Children, CANADA

## Abstract

**Background:**

For more accurate estimation of the global burden of pregnancy associated disease, clarity is needed on definition and assessment of non-severe maternal morbidity. Our study aimed to define maternal morbidity with clear criteria for identification at primary care level and estimate the distribution of and evaluate associations between physical (infective and non-infective) and psychological morbidities in two different low-income countries.

**Methods:**

Cross sectional study with assessment of morbidity in early pregnancy (34%), late pregnancy (35%) and the postnatal period (31%) among 3459 women from two rural communities in Pakistan (1727) and Malawi (1732). Trained health care providers at primary care level used semi-structured questionnaires documenting signs and symptoms, clinical examination and laboratory tests which were bundled to reflect infectious, non-infectious and psychological morbidity.

**Results:**

One in 10 women in Malawi and 1 in 5 in Pakistan reported a previous pregnancy complication with 1 in 10 overall reporting a previous neonatal death or stillbirth. In the index pregnancy, 50.1% of women in Malawi and 53% in Pakistan were assessed to have at least one morbidity (infective or non-infective). Both infective (Pakistan) and non-infective morbidity (Pakistan and Malawi) was lower in the postnatal period than during pregnancy. Multiple morbidities were uncommon (<10%). There were marked differences in psychological morbidity: 26.9% of women in Pakistan 2.6% in Malawi had an Edinburgh Postnatal Depression Score (EPDS) > 9. Complications during a previous pregnancy, infective morbidity (p <0.001), intra or postpartum haemorrhage (p <0.02) were associated with psychological morbidity in both settings.

**Conclusions:**

Our findings highlight the need to strengthen the availability and quality of antenatal and postnatal care packages. We propose to adapt and improve the framework and criteria used in this study, ensuring a basic set of diagnostic tests is available, to ensure more robust assessment of non-severe maternal morbidity.

## Introduction

Maternal mortality is sometimes referred to as ‘the tip of the iceberg’, sitting above a poorly documented and understood mass of morbidity. Whilst the causes of maternal mortality and morbidity may not be simply connected, it is recognised that many women suffer both long and short-term consequences of pregnancy delivery and the postpartum period, the major burden of which affects women living in developing countries [1–3].

It has been suggested that for every woman who dies, 20 or 30 suffer morbidity that is related to pregnancy and childbirth [4,5]. Annually, up to 20 million women worldwide are estimated to suffer morbidity related to pregnancy and childbirth [6].

Reproductive morbidity has been defined as ‘any morbidity or dysfunction of the reproductive tract, or any morbidity which is a consequence of reproductive behaviour including pregnancy, abortion, childbirth, or sexual behaviour and may include those of a psychological nature’ [7]. A proposed definition of maternal morbidity developed recently is “any condition that is attributed to or aggravated by pregnancy and childbirth, which has a negative impact on the woman’s wellbeing” [8].

Reproductive ill health is estimated to account for 22% of the global disease burden among women of reproductive age (15–44 years) with pregnancy-related conditions dominating the burden of reproductive ill health especially in developing countries [9].

Research on maternal morbidity has tended to focus on severe and life threatening acute maternal morbidity (SAMM), also termed ‘maternal near miss’, which has now been well defined and generally is assessed at secondary or tertiary health care levels [10–14]. In contrast, morbidity which is not immediately life threatening, but which may be recognised at primary level by a health care provider or perceived as morbidity by the woman herself (because it impairs her wellbeing and is associated with adverse pregnancy outcome), is poorly documented [15–17].

A major difficulty has been the lack of a standard definition and methodology for assessment of non-life threatening maternal morbidity. Previous studies describing maternal morbidity each include one or several of a wide spectrum of conditions ranging from short-term major complications such as obstetric haemorrhage and eclampsia, to long-term conditions such as obstetric fistula or anaemia in varying combinations [18–21]. In addition, much of the information and evidence reported to date is from hospital-based small studies with reporting and selection biases or is found in the grey literature as reports and surveys. Thus, there is currently a lack of information and understanding regarding prevalence and burden of maternal morbidity that is not immediately life threatening and even less data on psychological morbidity associated with pregnancy, particularly in rural areas of developing countries in Asia and Africa.

Information on women’s well-being and morbidity, both physical and psychological during and after pregnancy is important. It should inform targeted, effective antenatal and postnatal care packages, pre-pregnancy education and inform and support implementation of policies concerned with improving maternal and newborn health. Information of this nature is also useful to be able to assess specific needs that are not currently addressed during “routine” antenatal and or postnatal care. In addition, morbidity, where this can be defined, will serve as a useful outcome measure for effectiveness of programmes.

This study, conducted in Malawi and Pakistan, had three specific purposes: 1) to arrive at a more concise description or definition of "maternal morbidity", including physical (infective and non-infective) as well as psychological morbidity, with clear criteria for identification of the cases that can be applied at primary care level; 2) to systematically estimate the extent and distribution of physical (infective and non-infective) and psychological morbidity in two different countries on different continents; and 3) to evaluate the associations between infective, non-infective and psychological morbidity.

## Methods

This was a community based cross sectional descriptive study using a newly developed and standardised structured questionnaire to assess morbidity during early pregnancy (first 20 weeks), late pregnancy (last 20 weeks) and in the postnatal period (up to 6 weeks) at the primary care level in rural populations in Malawi and Pakistan.

In Malawi, the survey was conducted in a rural community in the southern region—Chiradzulu district. Malawi is administratively divided into 28 districts within three regions. The health system in Malawi has three levels—primary or Health Centre level, secondary or Hospital level and a tertiary level with 2 teaching Hospitals in Lilongwe and Blantyre. In Chiradzulu district, 95–6% of women attend one of 4 rural health centres for antenatal care and are seen there by a local nurse-midwife who also provides postnatal care at the health centre [22,23]. There is no system of home visits for care delivery and all primary level maternal and newborn health care is provided at the health centres which are free and on average within a 5 kilometre range of women living in this community.

In Malawi, the age of consent at the time of the study was 16 years (for marriage) and 13 years for sexual intercourse. We checked the database for age: in Malawi, 6 girls who accessed care and were included in the study reported their age as 15 years. All other women/girls were aged 16 and over. In Malawi, all women, regardless of age, were seen at the point they accessed routine antenatal or postnatal care at one of the participating clinics. All women were given a full explanation regarding the purpose of the study in the local language and provided written consent. For minors, accompanied by a parent or guardian, signed consent was provided (signature or thumb-print) using a consent form in the local language in the presence of the guardian or parent. All consultations were undertaken by trained healthcare providers and no additional risks were identified.

In Pakistan, the survey was carried out in Rawalpindi District situated in the north-east of Pakistan. Pakistan is administratively divided—in descending order of size—into provinces, division, districts, sub-districts (Tehsils), and union councils. Each union council oversees about 10–15 villages and a population of approximately 10,000–15,000 and has a Basic Health Unit (BHU) which has a work force consisting of a primary care physician, a Lady Health Visitor (LHV), a vaccinator, mid-wives and Lady Health Workers (LHWs). LHWs are members of the local community who have completed secondary school and are trained to provide mainly preventive mother and child health care and education. Each LHW is responsible for a population of 1500 people in her catchment area. In Pakistan, up to 73% of women receive antenatal care at least once from a skilled provider [23].

In Pakistan, all married women aged 15 to 49 years, resident in the 5 Union Councils in Tehsil Gujar Khan who were pregnant or had given birth in the 6 weeks prior to commencement of the study were identified through a household survey and were invited to participate in the study. 1750 women were approached and 13 women did not participate (5 refused and 8 were not in the home when the LHW called).

In Pakistan, women are legally allowed to marry at 18. Seven minors were included in the study—six reported their age as 17 years and one as 16 years. In Pakistan, all women were interviewed in their home setting, verbal permission was obtained from the head of the household (including the parents of girls aged under 18 years) and all women regardless of age. In addition, signed consent (signature or thumb-print) was obtained using a consent form in the local language.

All questionnaires and assessments were conducted by nurse-midwives (Malawi) or Lady Health Workers (Pakistan) who had been trained to use the questionnaire.

The structured questionnaire was translated into the Urdu language in Pakistan. In Malawi, the English version was used.

The questionnaire included information on basic demographic characteristics, previous obstetric morbidity, and current morbidity. For current morbidity, all questions were grouped to reflect infective morbidity, non-infective morbidity and psychological morbidity.

For infective morbidity, women were asked about signs and symptoms of infections including malaria, urinary tract infections, syphilis or other Sexually Transmitted Infections (STI), tuberculosis and whether they were on any medication. Women in Malawi were also asked about their HIV status. Women seen during the postnatal period were assessed for postnatal infections such as perineal infection, mastitis, or breast abscess. To quantify the prevalence of non-infective morbidity, women were asked about signs and symptoms of haemorrhage, pre-eclampsia, anaemia, and other medical conditions such as nausea, vomiting, epilepsy, and asthma. In addition, women were asked about incontinence of urine or faeces after delivery.

In addition to a standard obstetric assessment and clinical examination, simple laboratory and clinical examinations available at the point of contact were:

### Malawi

Blood Pressure, temperature, urinalysis using a simple dipstix, haemoglobin using a battery-operated HemoCue^®^, a VDRL test for syphilis, HIV test and ultrasound assessment of gestational age (bi-parietal diameter).

### Pakistan

Examination of conjunctiva for anaemia and gestational age estimated from last menstrual period (LMP). LHWs see women when they become pregnant or relatively early in pregnancy and LMPs are recorded at that time. LHW are not able to measure blood pressure or take blood for haemoglobin measurement nor do they have any dipsticks to test urine. LHW can refer women to the nearest BHU to have these examinations.

The Edinburgh Postnatal Depression Scale (EPDS) was embedded in the questionnaire to identify psychological morbidity. The EPDS is a tool developed for screening postpartum and pregnant women in outpatient, home visiting settings, or at the 6–8 week postpartum examination [24]. This tool has been previously used and validated in the developing country setting [25].

In both settings, the questionnaires were first piloted among both antenatal and postnatal women. Women found the questions easy to answer and non-threatening. Minor adjustments in language were made. LHWs and nurse-midwives found the questions and assessments easy to carry out as these were on the whole similar to those used during routine antenatal or postnatal care in both settings.

In Pakistan, the majority of the women were interviewed by the LHW at their home (89%) and the rest at a Basic Health Unit (7%) or Health House (4%). In Malawi, all women were seen at one of four separate Health Centres by a nurse-midwife.

### Sample size calculation and analysis

In each country, for each phase of pregnancy, information was collected for a minimum of 500 women. This sample size had 80% power and a 95% confidence level to detect the prevalence of morbidity at less than 5%.

Data collected was double entered into a Microsoft Access database, with password protection. Data was identifiable by study number only. SPSS 22 was used to analyse the data. Simple descriptive statistics were calculated to estimate the incidence of specific morbidities in this population.

The proportion of women with each sign and symptom of morbidity was calculated for each stage of pregnancy. Signs and Symptoms were grouped to reflect specific disease conditions and categorised to reflect either infective or non-infective morbidity. We calculated the proportion of women who had only one, two, three or more infective or non-infective morbidities, and further analysed this by stage of pregnancy.

Significance testing was done with Pearson’s Chi Square, with p <0.05 set as statistically significant.

To analyse psychological morbidity, EPDS responses were scored 0, 1, 2, or 3 according to increased severity of the symptom. The total score was determined by adding together the scores for each of the 10 items. Validation studies have utilized various threshold scores in determining which women were positive and in need of referral. Cut-off scores range from 9 to 13 points [26]. For this study, we used a cut-off point of 9 to define psychological morbidity. The scale was evaluated for internal consistency and reliability using the Cronbach’s reliability alpha coefficient.

Morbidity was classified as infective (including malaria, HIV, tuberculosis, sexually transmitted infections, sepsis) or non-infective (including haemorrhage, anaemia, pre-eclampsia, incontinence, nausea and vomiting). To evaluate the relationship between psychological morbidity and infective morbidity, non-infective morbidity, previous pregnancy history of complications and poor outcomes, and descriptive factors such as age and parity, we conducted bivariate analyses using logistic regression. Odds ratios with accompanying 95% confidence intervals were calculated. We used multivariate logistic regression modelling to identify all factors that were independently and significantly associated with psychological morbidity, and to calculate their adjusted odds ratio with 95% Confidence Intervals (CI). All variables that were statistically significant in the bivariate analyses were included in the multivariate analysis, including descriptive variables, previous pregnancy morbidity and outcome, infective and non-infective morbidities. Separate multivariate models were created for each country. In creating the best logistic regression model, the Forwards Conditional methods were used, with variables remaining in the model only if p-values were less than 0.05 even with the addition of other variables. Models were evaluated using the -2 log likelihood ratio statistic and the Hosmer-Lemeshow goodness of fit test.

In Malawi, the study was approved by the College of Medicine Research and Ethics Committee (COMREC). (S1 Ethical Approval) In Pakistan, ethical approval was given by the institutional review board of the Human Development Research Foundation (IRB-HDRF). (S2 Ethical Approval)

## Results

### Study population

In Malawi, a total of 1732 questionnaires were completed, 34.3% in early pregnancy, 34.9% in late pregnancy and 30.8% at postnatal visit. In Pakistan, a total of 1727 women were interviewed, 33.8% in early pregnancy, 35.2% in late pregnancy and 31.0% at postnatal visit. General characteristics of the study population are given in Table 1.

**Table 1:** Characteristics of study population.

Characteristics	Number of women for which information available*	Early antenatal period*	Late antenatal period*	Postnatal period*	Total*
**Number of respondents (% of total)**	Malawi (n 1732)	592 (34.3)	605 (34.9)	535 (30.8)	1732
	Pakistan (n 1727)	584 (33.8)	608 (35.2)	535 (31.0)	1727
**Age (mean, sd, years)**	Malawi (n 1732)	24.7 (5.8)	24.4 (5.7)	24.3 (5.1)	24.5 (5.6)
	Pakistan (n 1726)	26.9 (5.2)	27.2(5.3)	27.9(5.1)	27.3 (5.2)
**Gravidity (median, min, max)**	Malawi (n 1730)	3 (1–8)	2.5 (1–10)	2.0 (1–8)	3.0 (1–10)
	Pakistan (n 1724)	2.0 (1–11)	3.0 (1–11)	3.0 (1–12)	3 (1–12)
**BMI (mean, sd)**	Malawi (n 1163)	21.8 +/- 3.2	22.9 +/- 3.3	22.8 +/- 3.1	22.5 +/- 3.3
	Pakistan (n 426)	22.7 +/- 5.2	24.1 +/- 4.6	25.0 +/- 6.0	24.0 +/- 5.3
**Gestational age at visit** **	Malawi (n 1068)	25.3 (4.8) weeks	32.8 (3.1) weeks	11.8 (6.3) days	NA
	Pakistan (n 1162)	15.9 (4.5) weeks	32.3 (4.2) weeks	13.4 (12.5) days	NA

* (number/ total reporting)

** in weeks or number of days post-delivery for postnatal women (mean, sd)

Similar to gravidity, median parity of the population was 2.0 in both settings with a maximum of 9 in Malawi and 12 in Pakistan. Mean (sd) height was 159.2 (7.0) cm in Malawi and 156.0 (13.4) cm in Pakistan. Weight was also similar; women in Malawi weighed on average (sd) 54.7 (7.9) kg in early pregnancy compared to 54.5 (10.1) kg in Pakistan and postnatal weight was recorded to be 55.9 (8.3) kg in Malawi and 57.6 (9.7) kg in Pakistan.

### Previous pregnancy morbidity and outcome

Overall, 10.4% of women in Malawi and 22.4% of women in Pakistan reported having had a complication during an earlier pregnancy. (Table 2) In both countries, the percentage of women reporting a previous complication increased with parity. The percentage of women who had been admitted to a health facility for complications in a previous pregnancy was much higher in Pakistan (13.8%) than in Malawi (2.7%) as was the proportion who had a previous CS (21.1% vs. 5.4%) A previous miscarriage or abortion was reported by more women in Pakistan where women register as being pregnant early compared to Malawi where women register a pregnancy much later (20.3% vs. 7.0%). Overall, 1 in 10 women in Malawi (9.5%) and Pakistan (11.4%) reported a previous neonatal death or stillbirth.

**Table 2:** Number and proportion of women who reported morbidity during previous pregnancy, by parity status.

% of women reporting morbidity	Country	1*	2*	3*	4*	> = 5*	%*
**Number of women**	Malawi	418	382	256	182	171	1409
	Pakistan	438	351	243	155	197	1454
**Previous Caesarean Section**	Malawi	6.7% (28/ 418)	7.3% (28/382)	3.1% (8/256)	1.6% (3/182)	5.3% (9/171)	5.4% (76/1409)
	Pakistan	25.3% (66/261)	25.1% (51/203)	17.2% (23/134)	10.6% (10/94)	18.0% (23/128)	21.1% (173/820)
**Previous neonatal death**	Malawi	2.9% (12/418)	4.5% (17/382)	7.4% (19/256)	9.3% (17/182)	9.4% (16/171)	5.7% (81/1409)
	Pakistan	2.1% (9/420)	5.5% (19/346)	8.8% (21/240)	8.4% (13/154)	15.8% (30/190)	6.8% (92/1350)
**Previous stillbirth**	Malawi	1.7% (7/418)	3.1% (12/382)	3.1% (8/256)	5.5% (10/182)	9.9% (17/171)	3.8% (54/1409)
	Pakistan	2.4% (10/ 420)	3.5% (12/347)	2.5% (6/240)	7.1% (11/154)	12.0% (23/191)	4.6% (62/1352))
**Previous abortion/Miscarriage**	Malawi	6.2% (26/418)	6.5% (25/382)	8.2% (21/256)	6.6% (12/182)	8.8% (15/171)	7.0% (99/1409)
	Pakistan	13.5% (59/ 438)	16.0% (56/351)	23.5% (57/243)	29.7% (46/155)	32.0% (63/196)	20.3% (281/1383)
**Complication in previous pregnancy**	Malawi	5.5% (23/418)	9.9% (38/382)	9.8% (25/256)	17.0% (31/182)	15.2% (26/171)	10.4% (143/1409)
	Pakistan	22.8% (97/425)	19.9% (70/351)	20.2% (49/243)	23.9% (37/155)	27.2% (53/ 195)	22.4% (306/1369)

* (number/ total reporting)

### Current Pregnancy Morbidity

#### a. Infective Morbidity

Infective Morbidity is categorised and presented in Table 3.

**Table 3:** Signs and Symptoms of Infective Morbidity.

Signs and symptoms		Early Pregnancy*	Late Pregnancy*	Postnatal*	Total*
**Malaria positive slide or treated for malaria**	Malawi	8.8% (52/592)	8.8% (53/ 602)	6.7% (36/534)	8.2% (141/1728)
	Pakistan	3.8% (22/584)	2.3% (14/607)	2.1% (11/530)	2.7% (47/1721)
**Fever of unknown origin** ^1^	Malawi	2.7% (16/592)	3.5% (21/604)	0.9% (5/535)	2.4% (39/1731)
	Pakistan	3.6% (21/584)	3.2% (20/608)	2.2% (12/535)	3.1% (53/1727)
**Mastitis or breast abscess**	Malawi			0.7% (4/535)	0.7% (4/535)
	Pakistan			3.0% (16/ 528)	3.0% (16/528)
**Signs and symptoms of a Urinary Tract Infection** ^2^	Malawi	4.7% (28/590)	6.2% (37/600)	5.2% (28/535)	5.4% (93/1725)
	Pakistan	8.8% (51/584)	9.2% (54/ 607)	6.8% (36/530)	8.2% (141/1721)
**Signs and symptoms of a Sexually Transmitted Infection** ^3^	Malawi	8.0% (47/590)	8.0% (48/601)	7.5% (40/533)	7.8% (135/1724)
	Pakistan	16.4% (96/584)	15.8% (96/607)	12.1% (64/530)	14.9% (256/1721)
**HIV positive or two or more HIV associated conditions** ^4^	Malawi	15.6% (92/591)	16.1% (97/602)	16.5% (88/534)	16.0% (277/1727)
	Pakistan	6.7% (39/584)	5.3% (32/607)	7.0% (37/530)	6.3% (108/1721)
**Tuberculosis or suspected tuberculosis** ^5^	Malawi	0.5% (3/590)	0.6% (4/602)	1.1% (6/535)	0.8% (13/1727)
	Pakistan	12.0% (70/584)	10.5% (64/607)	7.2% (38/530)	10.0% (172/1721)
**Hepatitis**	Malawi	0	0	0	0
	Pakistan	1.4% (8/580)	2.1% (13/ 607)	1.1% (6/530)	1.6% (27/1717)

^1^ Women reporting fever and/or chills in the last four weeks for whom it is not possible to define any particular infective morbidity using study criteria.

^2^ Dysurea and frequency

^3^ Vaginal or labial ulcer or inguinal bubo OR abnormal discharge and lower abdominal pain OR VDRL positive or had benzyl penicillin in this pregnancy or received treatment for STI.

^4^ Any two or more conditions associated with HIV positive status, or HIV positive.

^5^ Suspected tuberculosis associated with HIV positive status, or HIV positive.

* % (number/number assessed)

Between 6.8% and 10.3% of women in Malawi and Pakistan respectively reported a history of fever and feeling unwell in the month before they were seen, with more women reporting this in the antenatal compared to postnatal period. In Malawi, all women had their temperature checked and 0.8% had a fever (defined as >37.5° Celsius) at the time of visit. Using the criteria defined in this study we identified a possible infective cause of reported fever in the last month in all but 2.4% of women in Malawi and 3.1% of women in Pakistan.

Malaria was more common in Malawi than in Pakistan throughout pregnancy—with 8.1% of women in Malawi having been treated for malaria although only a maximum of 1.0% had been tested and had a positive malaria slide at time of visit. In Malawi, all pregnant women receive malarial prophylaxis as part of routine antenatal care. In Pakistan, no blood tests were conducted but 2.7% of women had received treatment for malaria and malaria prophylaxis is not part of routine antenatal care.

The percentage of women who had signs and symptoms of a sexually transmitted infection (using the syndromic approach) in Pakistan was much higher than in Malawi– 14.9% vs. 7.8%). This was at least in part attributable to the larger number of women reporting both vaginal discharge and lower abdominal pain in Pakistan (14.2% vs. 1.2% in Malawi).

The incidence of tuberculosis or suspected tuberculosis was estimated to be 10.0% in Pakistan. In Malawi and Pakistan assessment or screening for tuberculosis (TBC) (other than asking about cough and medication) is not routinely done during antenatal care (ANC) or postnatal care (PNC). Testing for HIV is not carried out in the routine ANC and PNC setting in Pakistan, while this is done in Malawi. We calculated the proportion of women with at least two of six conditions commonly associated with a HIV positive status: recurrent fevers, oral thrush, chronic diarrhoea, herpes zoster, chronic cough or weight loss. In Pakistan, just over 6.3% of all women reported at least 2 of these signs and symptoms. None of these women were on TBC treatment or had been test for HIV. In Malawi, HIV positive status was recorded by 15.4% of women, and an additional 0.6% of women who were not already known to be HIV positive reported at least two out of these six conditions associated with positive HIV status. In total, 38 women had at least two of these symptoms and of these 26 were known to be HIV positive.

Overall, 1 in 3 women reported at least one type of infective morbidity (32.6% in Malawi and 31.6% in Pakistan). Infective morbidity was evenly spread across the time period of pregnancy in Malawi, with about one third of the women having at least one infection throughout pregnancy and postnatal periods; 31.8% reported at least one infective morbidity in early pregnancy, 34.8% in late pregnancy and 31.2% in the postnatal period. In Pakistan, these percentages were significantly different between pregnancy and postnatal periods: 34.6%, 33.4% and 26.2%, p = 0.005. The majority of women had just one identified infective morbidity, with less than 10% reporting multiple morbidities. (Table 4) In Malawi, further analyses revealed no difference between stage of pregnancy and burden of infective morbidity. However, in Pakistan, significant differences were noted across stage of pregnancy among women who report only one infective morbidity with less morbidity reported in the postnatal compared to the antenatal period (p <0.01). This difference was no longer significant among women who reported two infective morbidities (p = 0.06) or three or more morbidities (p = 0.91).

**Table 4:** Burden of infective morbidity among pregnant and postnatal women in Malawi and Pakistan.

Percentage of women with infective morbidities		Early Pregnancy*	Late Pregnancy*	Postnatal*	Total*
**One infective morbidity**	Malawi	25.2% (149/592)	28.0% (169/604)	24.5% (131/535)	25.9% (449/1731)
	Pakistan^1^	21.9% (128/584)	24.1% (146/607)	16.8% (89/530)	21.1% (363/1721)
**Two infective morbidities**	Malawi	5.4% (32/592)	5.3% (32/604)	5.4% (29/535)	5.4% (93/1731)
	Pakistan^2^	8.4% (49/584)	5.6% (34/607)	5.3% (28/530)	6.4% (111/1721)
**Three or more infective morbidities**	Malawi	1.2% (7/592)	1.5% (9/604)	1.3% (7/535)	1.3% (23/1731)
	Pakistan	4.3% (25/584)	3.8% (23/607)	4.2% (22/530)	4.1% (70/1721)

^1^ p <0.01

^2^ p = 0.06; Pearson’s Chi Square

* % (number/total assessed)

#### b. Non-Infective Morbidity

Bleeding was reported more commonly in Pakistan compared to Malawi and in both groups post- or intra-partum bleeding was more common than bleeding during pregnancy. Heavy bleeding at delivery was reported by similar numbers: 5.4% in Malawi and 6.4% in Pakistan. However many more women in Pakistan had received a blood transfusion (5.4% vs. 0.8% in Malawi). (Table 5)

**Table 5:** Signs and symptoms of non-infective morbidity.

Signs and symptoms	Country	Early Pregnancy*	Late Pregnancy*	Postnatal*	Total*
**Antepartum haemorrhage**	Malawi	0.7% (4/592)	1.5% (9/ 605)	1.7% (9/ 535)	1.3% (22/1732)
	Pakistan	4.6% (27/584)	4.1% (25/608)	3.6% (19/535)	4.1% (71/1727)
**Intra or Post-Partum haemorrhage** ^1^	Malawi			5.6% (30/535)	5.6% (30/535)
	Pakistan			16.9% (89/528)	16.9% (89/528)
**Anaemia**	Malawi	38.4% (206/537)	41.1% (231/562)	35.5% (22/62)	39.5% (459/1161)
	Pakistan	37.8% (221/584)	39.3% (239/608)	28.0% (150/535)	35.3% (610/1727)
**Pre-Eclampsia** ^2^	Malawi	0	0.2% (1/547)	0	0.2% (1/547)
	Pakistan	0.5% (3/584)	1.3% (8/608)	0.6% (3/535)	0.8% (14/1727)
**Incontinence of urine or faeces**	Malawi	0	0	0.9% (5/535)	0.9% (5/535)
	Pakistan	0	0	4.7% (25/527)	4.7% (25/527)
**Nausea and Vomiting**	Malawi	17.8% (105/590)	18.0% (108/600)	21.9% (117/535)	19.1% (330/1725)
	Pakistan	30.2% (176/583)	13.7% (83/607)	12.1% (64/529)	18.8% (323/1719)
**Epilepsy**	Malawi	0 (2/589)	0.2% (1/600)	0	0.3% (3/1189)
	Pakistan	1.2% (7/583)	0.5% (3/607)	0.8% (4/529)	0.8% (14/1719)
**Asthma**	Malawi	1.4% (8/591)	0.8% (5/602)	0.9% (5/535)	1.0% (18/1728)
	Pakistan	1.9% (11/583)	1.3% (8/607)	0.9% (5/529)	1.4% (24/1719)

^1^ Reported heavy bleeding at delivery or in postnatal period or received a blood transfusion

^2^ Preeclampsia defined in Malawi as (BP > 140/90 or on antihypertensive treatment) and proteinuria. In Pakistan preeclampsia defined as (BP>140/90 or on antihypertensive treatment or known to be hypertensive) and proteinuria.

* % (number/ total assessed)

Although anaemia was diagnosed using more accurate tools in Malawi (HemoCue®) than in Pakistan (mostly conjunctiva inspection combined with symptoms of anaemia), in both settings around 40% of women were found to be anaemic. Symptoms associated with anaemia which were specifically assessed included heart palpitations, headaches, dizziness, breathlessness, and problems carrying out daily work because of tiredness. In Pakistan, 50% of women reported at least two such symptoms; more in the antenatal (55.2%) compared to the postnatal period (38.2%). In Malawi, about one in four women (23.9%) reported two or more of these symptoms again more commonly in the antenatal (26.6%) than postnatal period (17.8%).

The occurrence of pre-eclampsia was difficult to establish in Pakistan as the LHW who sees the woman both for antenatal and postnatal care does not measure BP or check urine using a dipstick but has to refer for this to be done. In Pakistan, we considered a woman to have high blood pressure if she met any of the following criteria; 1) BP measures and above 140/90 (96/167 women), 2) reported by the LHW to be known hypertensive or, 3) on antihypertensive medication. Proteinuria and hypertension were noted in a maximum of 1.3% women in later pregnancy. If we included women with signs and symptoms associated with pre-eclampsia including headache, blurred vision, and upper abdominal pain, an additional 33 cases were identified early in pregnancy, 18 in late pregnancy and 18 in the postpartum period giving an overall estimated occurrence of pre-eclampsia of 5.4% (74/1727).

In Malawi, no additional cases were identified using this approach. Both tests (BP and urinalysis) are routinely carried out by the attending nurse-midwife providing ante and postnatal care. In Malawi, while high blood pressure was recognised to be present in up to 4.1% of women in late pregnancy and proteinuria present in up to 11.6% of women, both high blood pressure and proteinuria were documented in only 0.2% of women.

Incontinence (of urine or faeces) after delivery was reported by less than 1% of women in Malawi and around 5% in Pakistan.

One in five women reported nausea and vomiting in both settings and this was more common in early pregnancy especially in Pakistan where women are seen very early in pregnancy by the LHW.

Including nausea and vomiting, between 40% and 50% of women in Malawi and Pakistan respectively reported at least one non-infective morbidity with the majority identified to have one morbidity only. When nausea and vomiting are excluded, just less than one in three women (30.1%) in Malawi reported at least one non-infective morbidity: 38.4% in early pregnancy, 39.5% in late pregnancy and 11.6% in the postnatal period (p <0.01). In Pakistan this decreased to a little more than 40% of the women who reported at least one non-infective morbidity: 42.8% in early pregnancy, 42.7% in late pregnancy and 44.3% in the postnatal period, with no statistically significant difference across the three groups (p = 0.82). The majority of women reported only one non-infective morbidity with less than 5% in Malawi, and less than 10% in Pakistan reporting multiple non-infective morbidity. In Malawi, significant differences were noted across pregnancy stage among women reporting only one non-infective morbidity, with morbidity lowest in the postnatal period (p <0.001); no statistically significant differences were noted for those reporting two or more non-infective morbidities. In contrast, differences across pregnancy stage were not significant in Pakistan among women reporting only one non-infective morbidity, but these differences were noted for women with two (p = 0.02) or three or more non-infective morbidities (p = 0.04). (Table 6)

**Table 6:** Burden of non-infective morbidity among pregnant and postnatal women in Malawi and Pakistan.

Percentage of women with non-infective morbidities	Country	Early Pregnancy*	Late Pregnancy*	Postnatal*	Total*
**One non-infective morbidity**	Malawi^a^	35.5% (210/592)	38.2% (231/605)	10.8% (58/535)	28.8% (499/1732)
	Pakistan	34.1% (199/584)	36.7% (223/608)	32.1% (172/535)	34.4% (594/1727)
**Two non-infective morbidities**	Malawi	1.7% (10/592)	1.3% (8/605)	0.6% (3/535)	1.2%
	Pakistan^b^	7.4% (43/584)	4.8% (29/608)	8.8% (47/535)	6.9% (119/1727)
**Three or more non- infective morbidities**	Malawi	0	0	0.2% (1/535)	0.2% (1/535)
	Pakistan*	1.4% (8/584)	1.2% (7/608)	3.0% (16/535)	1.8% (31/1727)

* % (number/total assessed)

^a^ p<0.001

^b^ p<0.05; approaches significance

#### c. Psychological Morbidity

**Fig 1:**
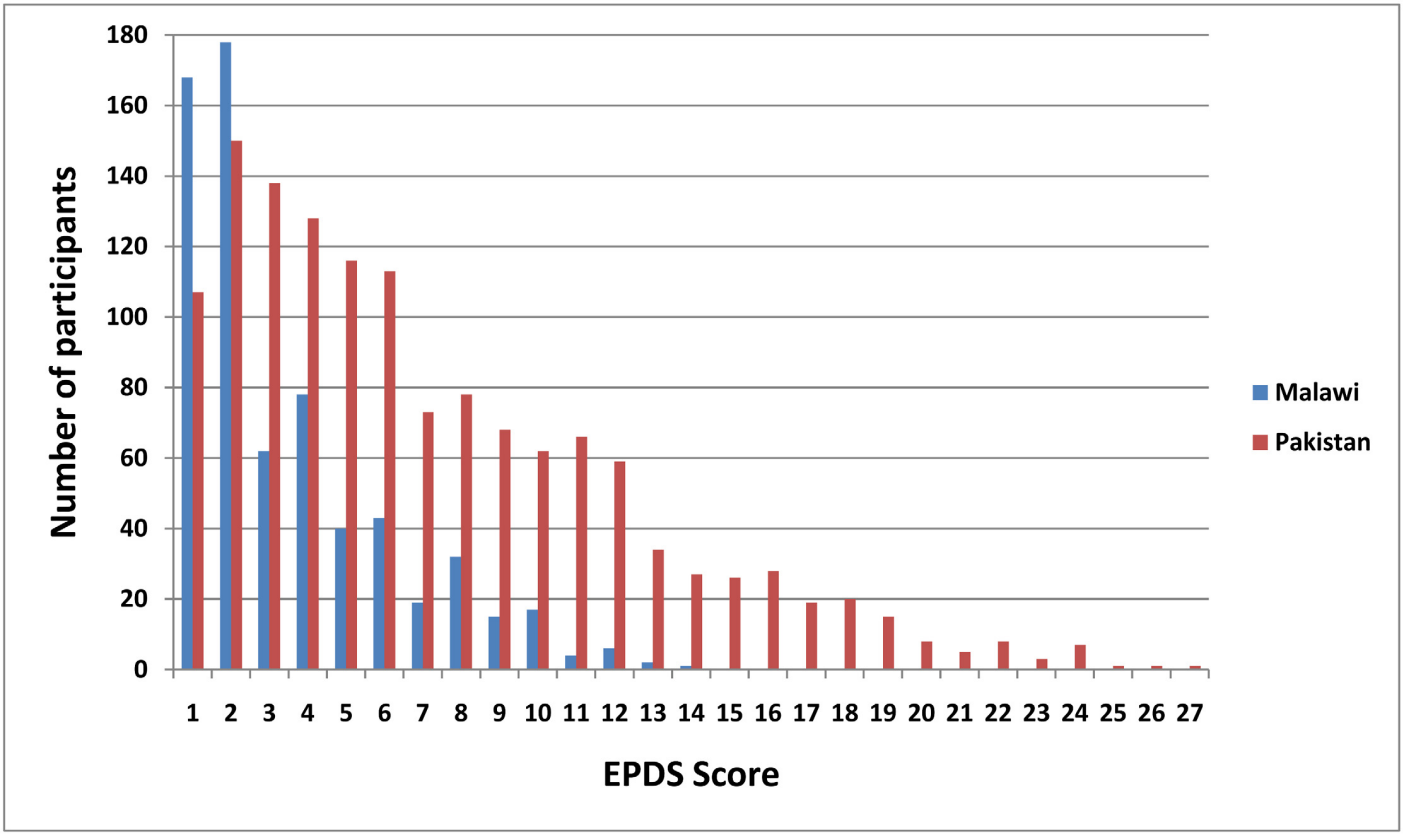
Distribution of EPDS scores for rural women in Malawi and Pakistan.

The EPDS performed well in both countries, with Cronbach’s reliability alpha coefficient of 0.80 in Pakistan and 0.71 in Malawi. Women in Pakistan generally reported higher severity of psychological morbidity compared to women in Malawi. In Malawi, nearly two thirds of the women did not report any psychological morbidity (EPDS score 0) and the mean (sd) EPDS score was 3.5 (2.7) among those who scored positive for at least one question. In Pakistan, only 20% had an EDPDS score of 0 with a mean (sd) of 7.2 (5.1) among those who scored positive for at least one section. (Fig 1).

Using a cut-off EPDS score of 9 or above, 26.9% of women in Pakistan (458/1705) and 2.6% in Malawi (45/1728) reported psychological morbidity.

In Malawi, the proportion of women with psychological morbidity (EPDS score > 9) increased significantly across the stages of pregnancy (1.2%, 3.2%, 3.6%, p = 0.02). In Pakistan, however, this trend was not significant (25.8%, 26.9%, 28.1%; p = 0.69), with around one in four women in Pakistan reporting psychological morbidity throughout pregnancy.

#### d. Association between Infective, non-Infective, and Psychological Morbidity

Overall, 53% of women in Pakistan and 50.1% in Malawi reported at least one morbidity (infective or non-infective). In Malawi, 11.4% (197/1732) and Pakistan 21.5% (372/1727) of women were identified to have both infective and non-infective morbidity.

In both countries, significant increases in odds of psychological morbidity were noted for women who had complications during a previous pregnancy while previous experience of a stillbirth or neonatal death (alone) was not associated with increased odds of psychological morbidity. (Table 7)

**Table 7:** Association between psychological morbidity, infective morbidity, non-infective morbidity and previous pregnancy outcome.

Previous pregnancy outcome	Country	OR	95% CI	p-value
Complication in previous pregnancy	Malawi	2.87	1.35–6.08	**0.006**
	Pakistan	2.0	1.55–2.59	**<0.0001**
Complication in previous postnatal period	Malawi	2.92	1.12–7.64	**0.03**
	Pakistan	1.56	1.23–1.98	**<0.0001**
Previous stillbirth or neonatal death	Malawi	1.52	0.59–3.92	0.39
	Pakistan	1.32	0.98–1.79	*0*.*07*
**Infective Morbidity**				
Malaria	Malawi	2.13	0.93–4.86	0.07
	Pakistan	2.57	1.43–4.63	**0.002**
UTI signs and symptoms	Malawi	8.14	4.11–16.11	**<0.0001**
	Pakistan	2.93	2.06–4.17	**<0.0001**
STI signs and symptoms	Malawi	3.09	1.46–6.56	**0.003**
	Pakistan	3.2	2.44–4.23	**<0.0001**
HIV Positive^1^	Malawi	5.58	2.96–9.80	**<0.0001**
	Pakistan	3.63	2.43–5.43	**<0.0001**
**Non-infective Morbidity**				
Antepartum Haemorrhage	Malawi	7.67	2.81–20.91	**<0.0001**
	Pakistan	1.44	0.81–2.39	0.16
Intra or Postpartum Haemorrhage	Malawi	4.38	1.28–15.01	**0.02**
	Pakistan	1.60	1.08–2.66	**0.02**
Anaemia	Malawi	0.79	0.39–1.60	0.51
	Pakistan	3.15	2.52–3.93	**<0.0001**
Pre-Eclampsia	Malawi	0	-	1.0
	Pakistan	3.13	2.05–4.77	**<0.0001**
Incontinence of Urine or Faeces	Malawi	0.0	—	1.0
	Pakistan	2.17	0.98–4.81	*0*.*06*
Nausea and Vomiting	Malawi	1.57	0.80–3.08	0.19
	Pakistan	1.39	1.07–1.81	0.14

^1^ In Malawi, HIV positive individuals are those who tested positive or had 2 or more signs and symptoms of HIV, while in Pakistan, HIV positive individuals are those with 2 or more signs and symptoms of HIV

For infective morbidity having signs and symptoms of a UTI, STI or being HIV positive or having signs and symptoms associated with HIV positivity were all independently significantly associated with psychological morbidity (Table 7) with an overall doubling of the odds of reporting psychological morbidity in Pakistan and trebling in Malawi with any identified morbidity. (Pakistan OR: 1.91; 95%CI 1.69, 2.17; p <0.0001; Malawi OR: 2.89; 95% CI 2.17, 3.83; p <0.0001)

Results of non-infective morbidity in Pakistan show significant increases in odds for women with intra and postpartum haemorrhage but not for antepartum haemorrhage. In Malawi, results show significantly increased odds for women reporting any type of haemorrhage. Pre-eclampsia and anaemia were significantly associated with increased psychological morbidity in Pakistan but not in Malawi. And while increasing burden of non-infective morbidity more than doubles the odds of high psychological morbidity among pregnant women in Pakistan (OR: 2.24; 95% CI 1.93, 2.61; p<0.0001), this was not significant in Malawi (OR: 1.39; 95%CI 0.80, 2.42; p = 0.24)).

In Malawi, significant increases in odds of psychological morbidity were found with increasing age (OR: 1.11, 95% CI 1.06, 1.16; p <0.0001) and parity (OR: 1.43; 95% CI 1.22, 1.67; p <0.0001). In Pakistan, no differences were noted by age (p = 0.56), and while a slight increase in odds were noted for parity, this only approached statistical significance (p = 0.07).

Multivariate logistic regression show that for Malawi, after controlling for parity and pregnancy stage, antepartum bleeding increased the odds of psychological morbidity 5-fold (OR: 5.01; 95% CI 1.60, 15.70; p = 0.006), while increasing burden of infective morbidity (i.e. for each additional infective morbidity) showed slightly more than 2.5 fold increase in the odds of having psychological morbidity (OR: 2.58; 95% CI 1.92, 3.47; p = 0.000). No other factors were significant.

For Pakistan, results show a 56% increase in odds of psychological morbidity due to increasing burden of infective morbidity (OR: 1.56; 95% CI 1.36, 1.79; p = 0.000), and a 78% increased odds due to increasing burden of non-infective morbidity (OR: 1.78; 95% CI 1.51, 2.11; p = 0.000), when controlling for the effect of complication during a previous pregnancy. No other factors were important.

## Discussion

In calculating the overall burden of ill-health associated with pregnancy and childbirth, it is important to define and estimate the incidence of pregnancy associated morbidity. Assessment of the extent of maternal morbidity is also an important step to ensure appropriate health care intervention packages are in place. For more accurate estimation of the global burden of maternal morbidity, clarity is needed on definition, assessment must be at population level and self-reported morbidity may need to be validated [1,2,17].

For the first time we have applied a standardised model and criteria for assessment of non-life threatening maternal morbidity which can be applied in a primary care setting as part of existing ante and postnatal care packages, and which addresses a major component along the continuum of maternal health. Using this method, more than half the women in Pakistan and Malawi were identified to have morbidity including 1 in 3 with infective and a similar proportion with non-infective morbidity. Very few women (less than 10%) were identified to have multiple morbidities in either category. By bundling reported symptoms and signs together with basic clinical examinations and laboratory tests this study confirms that women in low and middle income countries are likely to have a high burden of morbidity during pregnancy and the postpartum period. It may not be possible to confirm or refute specific conditions or disease in many cases given the absence of more precise diagnostic testing. However, the prevalence of anaemia in the women in our study in Malawi (40%) is similar to that reported in the Demographic Health Survey in Malawi in 2010 (38%) [27].

The earliest reported estimate of maternal morbidity is from a prospective study among 280 women in rural India who were followed for 5 years and visited monthly. This study reported pregnancy related morbidity in 30% of women [28]. A larger cross sectional community based study from India among women aged less than 35 years with at least one child under 5 years old found that 41% of women had experienced morbidity during the last pregnancy [29]. Postpartum morbidity was assessed by trained village workers in India in the first 28 days after delivery and estimated to occur in up to 42.9% of women [30].

A number of other community based surveys have remained unpublished: in 1995, a cross sectional survey of 7325 households across one Governate in Egypt reported the burden of maternal morbidity based on women’s self-reported symptoms [31]. Overall, 82.8% of women reported morbidity in the antenatal period, 20.4% intrapartum, and 19.1% postpartum. The majority of women perceived these as serious health problems (80.3%). A similar study from Uganda in 1993 among 1261 women in 12 districts reported that 10.6% of women reported signs and symptoms of pre-eclampsia and eclampsia. Fever (44.9%), excessive headaches (26.9%), severe vomiting (19.6%), anaemia (19.0%) were the most frequently reported pregnancy related morbidities among women in Uganda [32]. Other authors have suggested an ‘enormous but unaddressed problem shrouded in a culture of silence and endurance’ [3,7,33]. The limitations of self-reporting are well recognised but it could be argued that these are signs and symptoms which women themselves consider as significantly contributing to ‘non-health’ or associated with adverse pregnancy outcome.

In our study, we have supplemented women’s reported signs and symptoms with simple clinical examination and diagnostic tests that are usually available in the antenatal and postnatal care settings in low and middle income settings.

The methodology we propose for identification of morbidity was found to be easy to apply and very acceptable in the primary care setting in both Malawi and Pakistan but the robustness with which an exact diagnosis of morbidity could be made relied on available clinical and laboratory examinations. These are currently still very limited in most primary care settings in developing countries even though this is where the majority of women receive ante and postnatal care and despite clear guidelines regarding content of antenatal care packages [34,35]. Simple diagnostic technology such as a battery operated HemoCue® or Haemoglobin Colour Scale to measure haemoglobin and urine dipsticks to measure proteinuria should be available in these settings [36]. The competence of the health care worker providing ante and postnatal care also needs to be taken into consideration, for example, it can be argued that any health care provider at primary care level who is expected to provide antenatal and postnatal care should be able to measure blood pressure and we note that this is currently not the case in a number of settings especially in Asia. Testing for HIV and syphilis requires minimal infrastructure and equipment and are both part of the core WHO antenatal care package. We note that between the two settings in this study practice was different; in Pakistan care was provided in or close to a woman’s home setting whereas in Malawi this was in a primary care health centre setting. However this also had implications for the level of clinical examination and diagnostic testing that was possible. Finally, where morbidity is recognised and identified it is important that health care providers have a clear pathway for further diagnostics, treatment or referral as needed. The findings of this study highlight the need to strengthen the ‘enabling environment’ and to improve the availability, content and quality of ante and postnatal care at primary care level in developing country settings.

A major contribution of this study is its evaluation of psychological morbidity as part of a comprehensive assessment of morbidity during pregnancy and the postpartum period. We found a remarkable difference between reported psychological morbidity in Pakistan where 1 in 4 women reported signs and symptoms of psychological morbidity whereas this was reported by less than 5% in Malawi. This may in part be due to differences in the way that data were collected—more private home setting in Pakistan compared to the more public less intimate, health centre in Malawi which could negatively affect disclosure. In addition, women in Malawi are generally active in agricultural or other work and not confined to the home environment as much as women in Pakistan might be. Finally, in many African settings, admitting to problems or feelings of anxiety or worry may be associated with ‘wishing this upon oneself’ limiting the use of a tool such as the Edinburgh Postnatal Depression Scale (EPDS). Thus, there is likely to be a variety of cultural and social practices and beliefs surrounding pregnancy which contribute to or prevent anxiety, depressed mood, and depression as well and determine if and how a woman can express this. A recent systematic review of perinatal mental health in pregnancy in Africa reported prevalence rates not dissimilar to women in developed countries but ranging between 4.3% and 17.4% during pregnancy and 3.2% to 48.0% postnatally [37]. In Pakistan, an earlier study using a combination of assessment tools identified 36% of postnatal women with high EPDS scores (>12) in the postnatal period [38]. The EPDS is the most widely used screening tool for postpartum depression and has also been extensively used in the antenatal period. A systematic review of validation studies reported a wide variety of cut off points and highlighted that this tool may not be equally valid across all settings [26]. A study from Ethiopia suggested limited validity of the EPDS in rural low income sub Saharan Africa compared to more urban populations [39]. It is widely recognised that there are a variety of questionnaires, including the EPDS, which examine depressive mood and are not diagnostic measures per se and most previous studies in Africa used a two phase approach: a questionnaire followed by clinical examination [36]. Although in this study the EPDS displayed good internal reliability in both countries (using Cronbach’s alpha score), studies which use more detailed interviews to diagnose depression may be required. Further research is needed to explore the reasons for these differences in psychological well-being including more exploratory and qualitative research methodology.

This was a cross sectional study and we were not able to assess if reported morbidity in the index pregnancy was associated with adverse pregnancy outcome. This would need further prospective studies in a variety of settings using a framework and criteria for assessing morbidity that is acceptable and applicable at population level. Assessments of maternal morbidity will need to be carried out in a wide variety of settings in low and middle income countries to capture the true burden of disease. We propose to adapt and improve the framework and criteria used in this study, ensuring a basic set of diagnostic tests is available to ensure more robust assessment of morbidity.

Maternal mortality has often been considered the primary indicator of maternal health. If maternal morbidity can be defined and assessed this could serve as an additional indicator which in particular will be useful to help evaluate effect of shorter term intervention programmes or those that cannot cover the large population groups needed to measure effect on mortality. It will be useful to be able to divide the morbidity–mortality spectrum into meaningful and defined clinical ranges. In the US, where maternal morbidity is classified as a public health problem, the ‘Healthy People 2010’ includes maternal morbidity during labour and delivery as new indicator for maternal health and there are calls to expand this further to include ante and postnatal morbidity [40].

As clinical decision making and care improves across this spectrum, maternal outcomes can be expected to improve [41]. By addressing both maternal morbidity and mortality, we can aspire to prevent not only unnecessary deaths but also complications of pregnancy.

## Supporting Information

S1 Ethical Approval Malawi: Ethical Approval from the College of Medicine Research and Ethics Committee (COMREC) (PDF)

S2 Ethical Approval Pakistan: Ethical Approval from the institutional review board of the Human Development Research Foundation (IRB-HDRF) (PDF)
