# Supplementary material for: Non-Life Threatening Maternal Morbidity: Cross Sectional Surveys from Malawi and Pakistan
Source: PLoS One. 2015 Sep 21;10(9):e0138026. doi: 10.1371/journal.pone.0138026 (PMC4577127; doi:10.1371/journal.pone.0138026)
Supplement: S2 Ethical Approval — (PDF) [file pone.0138026.s002.pdf]

Human
Development
Research
Foundation

Head Office:
House # 06, Street # 55, F-7/4, Islamabad.
Ph: 051-2656172-3
Po Box No. 759 G-10 Markaz, Islamabad.
Website: www.hdrfoundation.org
Email: info@hdrfoundation.org

Title: A Community Study of Maternal Morbidity And Domestic Violence In Rural Pakistan.

Principal Investigator: Prof Shamsa Rizwan

The committee of IRB-HDRF has considered the study and happy to approve this study to be conducted in Pakistan.

Abid Malik

Dr Abid Malik
Chairman IRB, HDRF 02.04.2008
