# Supplementary material for: Non-Life Threatening Maternal Morbidity: Cross Sectional Surveys from Malawi and Pakistan
Source: PLoS One. 2015 Sep 21;10(9):e0138026. doi: 10.1371/journal.pone.0138026 (PMC4577127; doi:10.1371/journal.pone.0138026)
Supplement: S1 Ethical Approval — (PDF) [file pone.0138026.s001.pdf]

UNIVERSITY OF MALAŴI

Principal

Prof. R.L. Broadhead, MBBS, FRCP, FRCPCH, DCH

College of Medicine
Private Bag 360
Chichiri
Blantyre 3
Malawi
Telephone: 677 245
677 291
Fax: 674 700
Telex: 43744

Our Ref.:

Your Ref.: COMREC/16

1st November, 2007

Dr Nynke van den Broek
College of Medicine
P/Bag 360
Blantyre 3

Dear Dr Broek

RE: P.05/07/559 – Survey of antenatal and postnatal morbidity in a rural population in Southern Malawi

I write to inform you that COMREC reviewed the above mentioned proposal which you resubmitted. I am pleased to inform you that COMREC approved your proposal on 1st November, 2007after considering that you addressed all the queries which were raised in an earlier review.

As you proceed with the implementation of your study I would like you to take note that all requirements by the college are followed as indicated on the attached page.

Sincerely,

Prof. E Borgstein
CHAIRMAN – COMREC

EB/tck
